# Recent improvement in survival outcomes and reappraisal of prognostic factors in hepatoblastoma

**DOI:** 10.1002/cam4.3897

**Published:** 2021-05-03

**Authors:** Kyung‐Nam Koh, Jung‐Man Namgoong, Hee Mang Yoon, Young Ah Cho, Se Hoon Choi, Juhee Shin, Sung Han Kang, Jin Kyung Suh, Hyery Kim, Seak Hee Oh, Kyung Mo Kim, Dae Yeon Kim, Ho Joon Im

**Affiliations:** ^1^ Divison of Pediatric Hematology/Oncology Department of Pediatrics Asan Medical Center Children’s Hospital University of Ulsan College of Medicine Seoul Korea; ^2^ Department of Pediatric Surgery Asan Medical Center Children’s Hospital University of Ulsan College of Medicine Seoul Korea; ^3^ Department of Radiology and Research Institute of Radiology Asan Medical Center University of Ulsan College of Medicine Seoul Korea; ^4^ Department of Thoracic and Cardiovascular Surgery Asan Medical Center University of Ulsan College of Medicine Seoul Korea; ^5^ Divison of Pediatric Gastroenterology, Hepatology and Nutrition Department of Pediatrics Asan Medical Center Children’s Hospital University of Ulsan College of Medicine Seoul Korea

**Keywords:** CHIC‐HS, hepatoblastoma, pediatric, prognostic factor, PRETEXT

## Abstract

**Background:**

Prognostic factors in hepatoblastoma need to be reevaluated considering the advances in treatment modalities. The study aimed to evaluate current outcomes of hepatoblastoma and reappraise the association of prognostic factors, including pre‐treatment extent of tumor (PRETEXT) stage with annotation factors and Children's Hepatic tumors International Collaboration‐Hepatoblastoma Stratification (CHIC‐HS) system, with survival outcomes.

**Methods:**

We evaluated 103 consecutive patients with hepatoblastoma retrospectively according to the treatment period based on the introduction of a liver transplantation program.

**Results:**

The 5‐year overall survival (OS), event‐free survival (EFS), and transplant‐free survival rates were 80.2%, 74.2%, and 61.8%, respectively. EFS and OS were improved significantly from 58.6% to 81.6% (*P *= 0.024) and from 58.6% to 90.8% (*P *< 0.001), respectively, in the late period (N = 74) compared with the early period (N = 29). The PRETEXT stage was significant or marginally significant for EFS and OS in the early period but not in the late period. The P, F, R, and C factors were significant for OS and EFS in the early period. However, in the late period, only the P factor was significant for OS, and the F and M factors were significant for EFS. The CHIC‐HS system was significant or marginally significant for EFS in both the early and late periods; however, it was significant for OS only in the early period.

**Conclusion:**

Survival rates were significantly improved in children with hepatoblastoma, especially in those with advanced PRETEXT stages with positive annotation factors and in a high‐risk CHIC‐HS group. Prognostic factors had different clinical implications with evolved treatment modalities.

## Graphical abstract

Survival rates were significantly improved among children with hepatoblastoma, especially those with advanced PRETEXT stages with positive annotation factors due to advances in surgical techniques and the introduction of effective chemotherapy. Prognostic factors had different clinical implications with evolved treatment modalities.

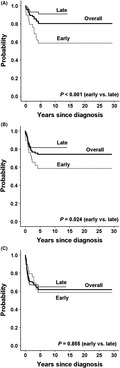

## 1 INTRODUCTION

Hepatoblastoma is the most common malignant liver tumor in children, and the estimated annual incidence is around 1.5 cases per million.^1^, ^2^ Treatment strategies incorporating chemotherapy and surgical resection have evolved over the past two decades, and these advances have significantly improved the survival outcomes, showing overall survival (OS) rates of more than 90% for low‐risk patients.^2^, ^3^, ^4^ However, there are still considerable differences in outcomes for low‐risk and high‐risk patients.^2^, ^5^, ^6^, ^7^

There are various known prognostic factors, such as the metastatic disease at diagnosis or pre‐treatment extent of tumor (PRETEXT) stage, histological subtypes, and serum alpha‐fetoprotein (AFP) levels.^2^, ^8^, ^9^, ^10^ In particular, the PRETEXT stage based on the anatomical extent of the primary tumor is known to be closely associated with the outcome as surgical resection of the primary tumor is the mainstay of treatment of hepatoblastoma.^10^, ^11^, ^12^

The PRETEXT stage can reflect surgical resectability with its detailed annotation factors. However, many clinical trial groups have used different staging systems with modifications in PRETEXT staging. Recently, the Children's Hepatic tumors International Collaboration (CHIC) collected extensive data from eight multicenter trials over 25 years, validated the prognostic significance of the PRETEXT stage with annotation factors, and proposed a new risk stratification scheme (CHIC‐Hepatoblastoma Stratification, CHIC‐HS) based on age, metastasis, and PRETEXT stage with annotation factors.^8^, ^13^

Recent advances in surgical techniques, such as indocyanine green (ICG) fluorescence‐guided surgical and especially liver transplantation (LT), have contributed to considerable improvements in the outcomes for advanced hepatoblastoma.^14^, ^15^, ^16^ Prognostic factors need to be reappraised, taking into account the advances in treatment modalities. Therefore, this study was conducted to reappraise the prognostic factors, including the PRETEXT stage with annotation factors, and to evaluate the effect of treatment advancement on the prognostic value of these factors. We also aimed to determine the clinical implication of the CHIC‐HS system in an actual clinical setting.

## 2 METHODS

### 2.1 Patients

From January 1991 to September 2019, 103 consecutive children with pathologically confirmed hepatoblastoma were treated at Asan Medical Center. The patients were retrospectively staged according to the PRETEXT staging system developed in 2005 by the Liver Tumor Study Group of the International Society of Pediatric Oncology (SIOPEL) group and revised in 2017.^17^, ^18^ In accordance with the 2017 PRETEXT staging system,^18^ PRETEXT stages with annotation factors were evaluated by two experienced pediatric radiologists (H.M.Y. and Y.A.C.) by consensus based on pretreatment CT or MRI scans. They were blinded to the clinical outcome.

The PRETEXT stages were grouped as follows according to the number of tumor‐free hepatic sections: PRETEXT I, three adjoining sections free from the tumor; PRETEXT II, two adjoining sections free from the tumor; PRETEXT III, one section free from the tumor; and PRETEXT IV, no tumor‐free sections. Annotation factors included the involvement of the hepatic vein (HV)/inferior vena cava (IVC) (V) and portal vein (PV) (P), the extrahepatic spread of disease (E), multifocality (F), tumor rupture (R), caudate involvement (C), lymph node metastases (N), and distant metastases (M). Annotation V and P were considered as positive if it met any of the following criteria: (a) The tumor obliterates all three first‐order HVs or the intrahepatic IVC for annotation V and either both first‐order PVs or the main PV for annotation P. (b) The tumor encases all three first‐order HVs or the intrahepatic IVC for annotation V and either both first‐order PVs or the main PV. (c) There is tumor thrombus in any one (or more) first‐order HVs or the intrahepatic IVC for annotation V and either or both the right and left PVs, or the main PV. Annotation R was defined as free fluid in the peritoneal space with imaging evidence of bloody components or presence of visible rupture. In addition, aggregate factors of VPEFR, defined as the presence of one or more of the V, P, E, F, or R factors, and VPEFR2, defined as the presence of two or more of these factors, were assessed.

The CHIC‐HS risk stratification system previously proposed by the CHIC was also applied retrospectively to the patients.^8^

### 2.2 Treatment

Patients with resectable disease at the time of presentation underwent surgery based on the principle of total tumor excision. Patients with unresectable, borderline resectable, or metastatic disease were diagnosed pathologically by percutaneous needle biopsy or intraoperative incisional biopsy. They were treated with preoperative chemotherapy to reduce the tumor size to increase respectability.

The number of preoperative chemotherapy cycles was between 2 and 6, which was determined at the physician's and surgeon's discretion based on the tumor response to chemotherapy and the possibility of complete tumor resection. After surgery, the patients underwent at least two courses of postoperative chemotherapy.

Over the period of the study, three cisplatin‐based Children's Cancer Group (CCG)/Pediatric Oncology Group (POG)/Children's Oncology Group (COG) chemotherapy regimens were used. The cisplatin/doxorubicin (CD) regimen (based on the CCG‐823F trial) consisted of cisplatin (90 mg/m^2^) on day 1 and doxorubicin (20 mg/m^2^/day) on days 1 through 4. Treatment cycles were repeated every 21 days.^19^ The C5 V regimen (based on the INT‐0098 trial) consisted of cisplatin (100 mg/m^2^) on day 1, 5‐fluorouracil (600 mg/m^2^) on day 2, and vincristine (1.5 mg/m^2^) on days 2, 9, and 16. Treatment cycles were repeated every 21 days.^20^ From 2007, high‐risk patients were treated with the C5VD regimen (based on AHEP0731, modified by omitting vincristine and irinotecan window therapy), which consisted of cisplatin (100 mg/m^2^) on day 1, 5‐fluorouracil (600 mg/m^2^) on day 2, vincristine (1.5 mg/m^2^) on days 2, 9, and 16, and doxorubicin (30 mg/m^2^) on days 1 and 2. Treatment cycles were repeated every 21 days.^21^ High‐risk disease was defined as having distant metastases, PRETEXT IV, or PRETEXT III with the V, P, E, F, or R annotation factors, or PRETEXT II or higher with serum AFP levels ≤100 ng/mL.

### 2.3 Analysis of prognostic factors

As the LT program for hepatoblastoma was introduced in our center in 2006, patients were divided into two groups based on each patient's time of diagnosis (1991–2005 and 2006–2019, referred to as the early and late treatment periods, respectively). To analyze the prognostic factors, age at diagnosis, sex, serum AFP levels, metastasis, PRETEXT stage with annotation factors, and CHIC‐HS were evaluated according to the treatment periods.

### 2.4 Statistical analysis

Event‐free survival (EFS) was defined as the time from the diagnosis until the first relapse, disease progression, second malignancy, or death from any cause or last contact. OS was defined as the time from the diagnosis to death from any cause or last contact. Transplant‐free survival (TFS) was defined as the time from the diagnosis to LT or death from any cause.

Pearson's chi‐square test was used to compare the categorical variables between the early and late treatment cohorts. The Kaplan–Meier method was used to estimate survival probabilities, and the log‐rank test was used to test the prognostic significance of various risk factors. Values of *p *< 0.05 were considered statistically significant. All statistical analyses were performed using SPSS software (version 21.0; SPSS Inc., Chicago, IL).

## 3 RESULTS

### 3.1 Patient characteristics

The clinical characteristics of the 103 patients are summarized in Table 1. There were 53 males and 50 females. The median age at diagnosis was 17 months (range, 0–261 months). The median follow‐up was 98 months (range, 10–352 months). There were no statistical differences between the early and late treatment cohorts in terms of sex, age at the time of diagnosis, serum AFP levels, PRETEXT stage, metastasis, and pathological subtypes.

**TABLE 1:** Patient characteristics at diagnosis and treatment.

Characteristics	Overall (N = 103)	Early (N = 29)	Late (N = 74)	*p* value
Age at diagnosis, years				0.890
< 1	32 (31.1%)	9 (31.0%)	23 (31.1%)	
1–2	36 (35.0%)	11 (37.9%)	25 (33.8%)	
3–8	20 (19.4%)	6 (20.7%)	14 (18.9%)	
≥ 8	15 (14.6%)	3 (10.3%)	12 (16.2%)	
Sex				0.074
Male	53 (51.5%)	19 (65.5%)	34 (45.9%)	
Female	50 (48.5%)	10 (34.5%)	40 (54.1%)	
PRETEXT				0.254
I	6 (5.8%)	1 (3.4%)	5.0 (4.3%)	
II	41 (39.8%)	16 (55.2%)	25.0 (29.5%)	
III	34 (33.0%)	7 (24.1%)	27.0 (36.5%)	
IV	22 (21.4%)	5 (17.2%)	17.0 (23.0%)	
Pathology				0.109
Epithelial	74 (78.7%)	14 (63.6%)	60.0 (83.3%)	
Mixed epithelial and mesenchymal	14 (14.9%)	5 (22.7%)	9.0 (12.5%)	
Macrotubular	5 (5.3%)	3 (13.6%)	2.0 (2.8%)	
Small cell undifferentiated	1 (1.1%)	0	1.0 (1.4%)	
Missing	9	7	2	
Serum AFP concentration, ng/mL				0.412
< 100	2 (2.0%)	1 (3.6%)	1 (1.4%)	
100–999	2 (2.0%)	1 (3.6%)	1 (1.4%)	
1000–1,000,000	83 (84.7%)	21 (75.0%)	62 (59.3%)	
> 1,000,000	11 (11.2%)	5 (17.9%)	6.0 (7.9%)	
Missing	5	1	4	
Metastasis				0.258
No	69 (67.0%)	17 (58.6%)	52 (70.3%)	
Yes	34 (33.0%)	12 (41.4%)	22 (29.7%)	
Lung	33 (32.0%)			
Bone	3 (2.9%)			
Heart	1 (1.0%)			
Chemotherapy				< 0.001
CD	18 (17.5%)	15 (51.7%)	3 (4.1%)	
C5 V	52 (50.5%)	14 (48.3%)	38 (51.4%)	
C5VD	33 (32.0%)	0	33 (44.6%)	
Surgery				< 0.001
Upfront	8.0 (7.8%)	5 (17.2%)	3 (4.1%)	
Delayed resection	68 (66.0%)	18 (62.1%)	50 (67.6%)	
LT	19 (18.4%)	0	19 (25.7%)	
Not performed	8 (7.8%)	6 (20.7%)	2 (2.7%)	

Abbreviations: AFP, alpha‐fetoprotein; C5 V, cisplatin/5‐fluorouracil/vincristine; C5VD, cisplatin/5‐fluorouracil/vincristine; CD, cisplatin/doxorubicin; LT, liver transplantationPRETEXT, pre‐treatment extent of tumor.

According to the PRETEXT staging system, 6, 41, 34, and 22 patients had PRETEXT I, PRETEXT II, PRETEXT III, and PRETEXT IV diseases, respectively. A total of 34 patients (33.7%) had distant metastases at presentation, including 30 patients with lung metastases, 2 patients with both lung and bone metastases, 1 patient with both lung and heart metastases, and 1 patient with bone metastases. In particular, significantly less metastases were observed among patients under the age of 1 year at diagnosis (4/32, 12.5%) compared with patients in other age groups (16/36, 44.4% of patients between 1 and 2 years; 8/20, 40.0% of patients between 3 and 7 years; and 6/15, 40% of patients over 8 years) (*P *= 0.029). There was no difference in the distribution of PRETEXT stages according to age at the time of diagnosis.

### 3.2 Treatment course

**FIGURE 1:**
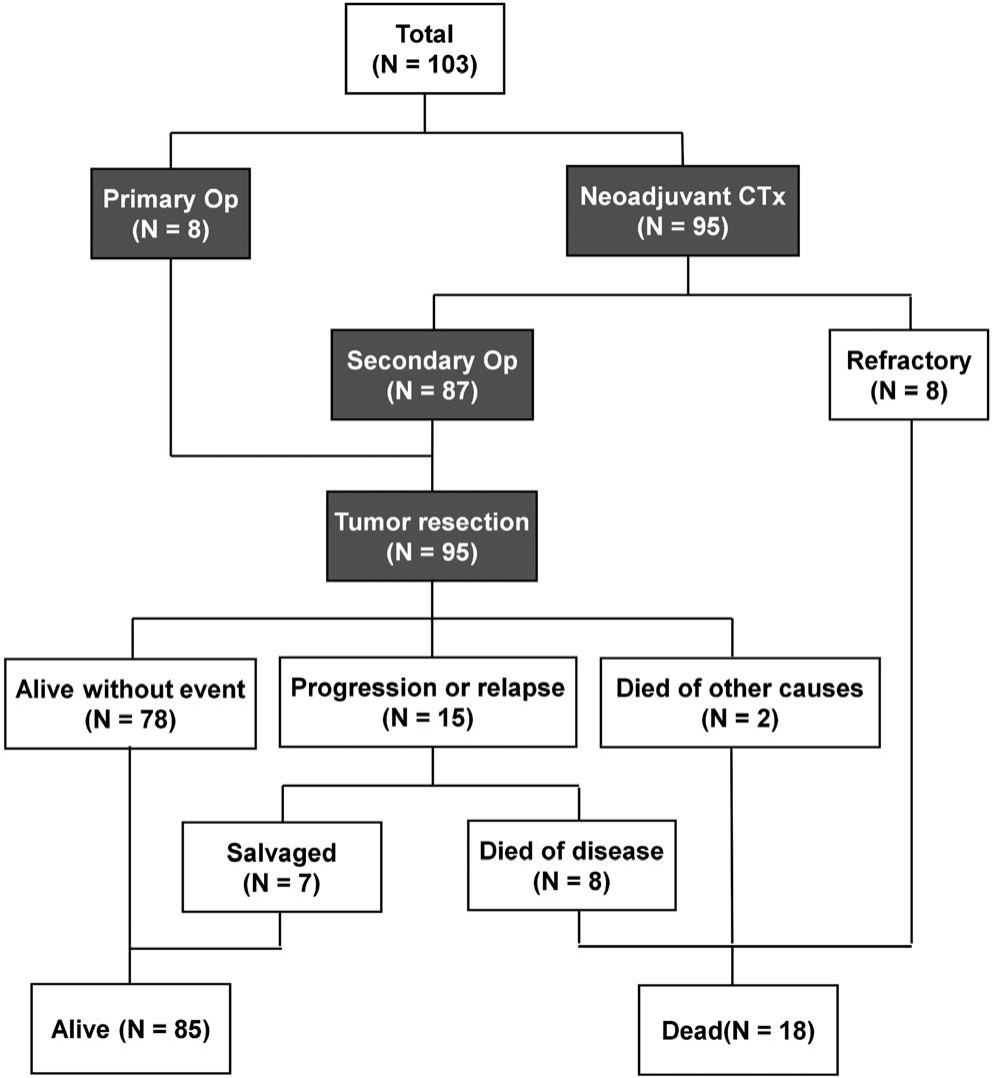
Clinical courses of 103 patients with hepatoblastoma. CTx, chemotherapy; Op, operation

The flow of the treatment course of the 103 patients is shown in Figure 1. Of the 103 patients, 8 patients (6.9%) underwent primary curative surgery, and the remaining 95 patients underwent biopsy, followed by preoperative chemotherapy. Another 87 patients underwent tumor resection (partial hepatic resection in 68 patients and LT in 19 patients), and 8 patients did not undergo surgery. ICG fluorescence‐guided resection was performed in 29 patients since 2016. Of the 95 patients who underwent surgery, 15 patients experienced progression or relapse, of which 7 patients were rescued by salvage therapy. Two patients died from causes not related to hepatoblastoma (one patient had therapy‐related myelodysplastic syndrome and one patient had underlying congenital adrenal hyperplasia). Finally, 85 patients were alive at the last follow‐up.

In particular, all 19 patients who underwent LT (including 4 patients with lung metastasis at diagnosis) after preoperative chemotherapy were alive. A total of 12 patients did not undergo chemotherapy after LT, and 7 patients underwent 1 or 2 cycles of chemotherapy after LT. Of the 12 patients who did not undergo postoperative chemotherapy, 2 patients relapsed in the lungs and were rescued with lung wedge resection and salvage chemotherapy.

Patients who successfully completed treatment, received a median four cycles of preoperative chemotherapy (range, 0–7) and a median two cycles of postoperative chemotherapy (range, 0–4). Postoperative chemotherapy was the same as preoperative chemotherapy, and the postoperative treatment plan did not change depending on the surgical specimen's tumor viability after chemotherapy.

Of 34 patients with metastasis at diagnosis, 14 patients underwent metastasectomy, and 11 of them survived. Among 20 patients who did not undergo metastasectomy, 7 patients died of refractory disease, and 13 patients achieved resolution of metastatic disease with chemotherapy alone and survived.

### 3.3 Overall outcome

**FIGURE 2:**
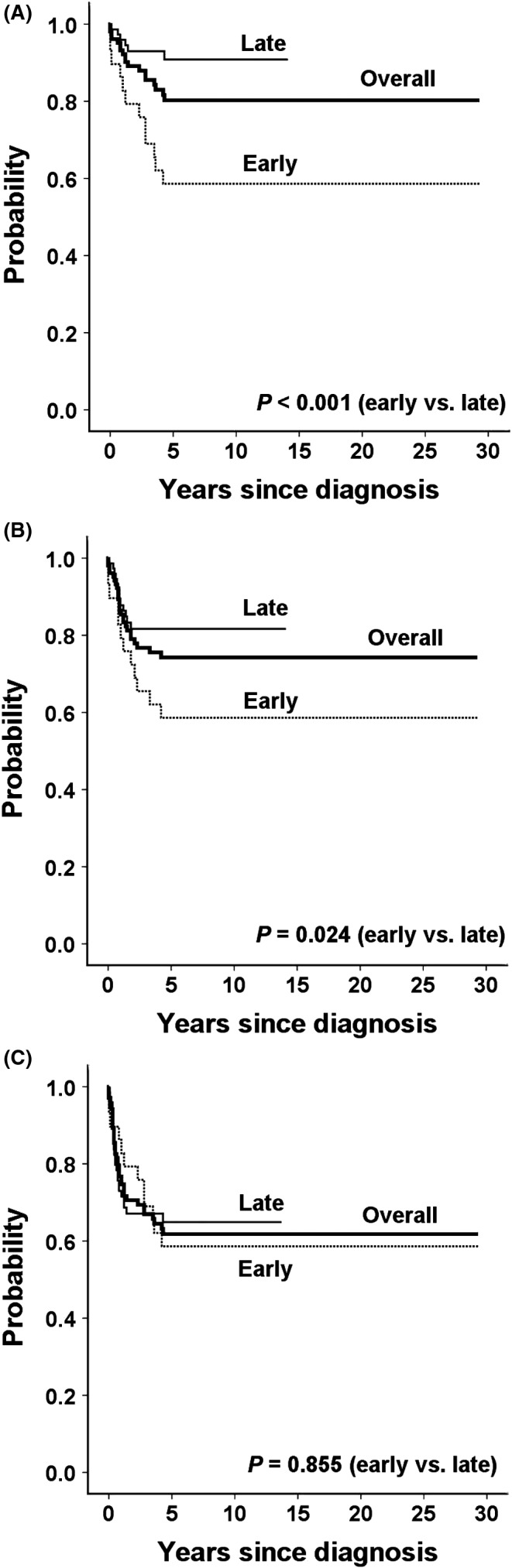
(A) Overall survival, (B) event‐free survival, and (C) transplant‐free survival rates for the overall, early, and late treatment cohorts of patients with hepatoblastoma

The OS and association of prognostic factors with survival outcomes according to the treatment period are presented in Table 2. The 5‐year OS, EFS, and TFS rates of 103 patients were 80.2%, 74.2%, and 61.8%, respectively, as shown in Figure 2. The EFS and OS rates were improved significantly in the late period (N = 74) compared with the early period (N = 29) (58.6% to 81.6%, *P *= 0.024 for EFS; 58.6% to 90.8%, *P *< 0.001 for OS). The 5‐year TFS rates were not different between the early and late periods (58.6% vs. 64.9%, *P *= .855).

**TABLE 2:** Prognostic factors and survival rates according to the treatment period.

Variable	5‐year OS									5‐year EFS						5‐year TFS					
	Overall			Early			Late			Overall		Early		Late		Overall		Early		Late	
	No.	%	*p*	No.	%	*p*	No.	%	*p*	%	*p*	%	*p*	%	*p*	%	*p*	%	*p*	%	*p*
Total	103	80.2		29	58.6		74	90.8		74.2		58.6		81.6		61.8		58.6		64.9	
Sex																					
Male	53	77.7	0.390	19	57.9	0.804	34	91.0	0.877	74.3	0.967	60.0	0.833	85.1	0.546	59.1	0.398	57.9	0.804	61.5	0.411
Female	50	83.2		10	60.0		40	90.9		74.3		57.9		78.7		64.8		60.0		67.7	
Age, years																					
< 1	32	91.8	0.292	9	88.9	0.019	23	90.9	0.210	89.5	0.025	88.9	0.023	88.9	0.043	78.9	0.040	88.9	0.019	71.9	0.284
1–2	36	72.4		11	27.3		25	96.0		69.9		27.3		91.4		53.8		27.3		68.0	
3–8	20	77.9		6	83.3		14	77.4		70.0		83.3		64.3		63.0		83.3		55.6	
≥ 8	15	78.8		3	33.3		12	91.7		60.0		33.3		66.7		45.7		33.3		50.0	
Pathology																					
Epithelial	74	86.7	0.340	14	64.3	0.800	60	93.2	0.124	79.0	0.720	64.3	0.776	82.8	0.489	63.7	0.553	64.3	0.800	64.7	0.658
Mixed	14	77.8		5	80.0		9	75.0		72.2		80.0		66.7		69.6		80.0		59.3	
Macrotubular	5	60.0		3	66.7		2	50.0		60.0		66.7		50.0		60.0		66.7		50.0	
SCU	1	100.0		0			1	100.0		100.0				100.0		0.0				0.0	
AFP at diagnosis																					
1^st^ tertile	32	93.8	0.046	9	88.9	0.053	23	95.7	0.601	90.4	0.012	88.9	0.059	90.9	0.182	75.0	0.268	88.9	0.053	73.9	0.739
2^nd^ tertile	33	73.5		9	33.3		23	91.1		69.5		33.3		82.1		56.4		33.3		60.9	
3^rd^ tertile	33	72.6		10	60.0		24	84.5		61.8		60.0		69.4		57.5		60.0		63.6	
NA	5																				
PRETEXT																					
1	6	100.0	0.440	1	100.0	0.054	5	100.0	0.445	83.3	0.103	100.0	0.039	80.0	0.350	100.0	<0.001	100.0	0.054	100.0	<0.001
2	41	83.9		16	68.8		25	96.0		81.6		68.8		91.6		83.9		68.8		96.0	
3	34	76.1		7	57.1		27	81.8		74.9		57.1		80.2		57.5		57.1		58.1	
4	22	74.4		5	20.0		17	94.1		58.4		20.0		70.1		15.2		20.0		17.6	
Chemotherapy																					
CD	18	72.2	0.186	15	66.7	0.397	3	100	0.711	72.2	0.712	66.7	0.414	100	0.717	55.6	0.409	66.7	0.391	0	<0.001
C5 V	52	76.4		14	50.0		38	88.0		71.0		50.0		80.3		66.3		50.0		74.2	
C5VD	33	93.7		0	NA		33	93.7		81.6		NA		81.6		60.5		NA		60.5	
V																					
No	67	83.8	0.102	18	66.7	0.137	49	92.5	0.382	78.9	0.077	66.7	0.160	84.9	0.279	70.7	0.004	66.7	0.137	74.2	0.018
Yes	36	74.0		11	44.5		25	87.6		65.7		45.5		75.6		46.0		45.5		47.7	
P																					
No	84	85.3	0.003	24	66.7	0.025	60	94.2	0.025	78.7	0.017	66.7	0.029	84.2	0.166	72.1	<0.001	66.7	0.025	75.7	<0.001
Yes	19	53.7		5	20.0		14	76.6		53.2		20.0		71.4		0.1		20.0		19.0	
E																					
No	99	79.8	0.461	29	58.6	NA	70	90.5	0.602	74.4	0.929	58.6	NA	82.3	0.723	62.4	0.346	58.6	NA	65.8	0.385
Yes	4	100.0		0	NA		4	100.0		66.7		NA		66.7		50.0		NA		50.0	
F																					
No	68	83.2	0.191	24	66.7	0.012	44	94.2	0.166	80.7	0.014	66.7	0.013	89.9	0.018	74.1	<0.001	66.7	0.012	80.2	<0.001
Yes	35	74.9		5	20.0		30	86.2		61.9		20.0		69.8		37.8		20.0		42.9	
R																					
No	96	81.1	0.410	27	63.0	0.007	69	90.1	0.483	76.8	0.041	63.0	0.013	83.6	0.258	63.4	0.189	63.0	0.007	65.2	0.866
Yes	7	68.6		2	0.0		5	100.0		42.9		0.0		60.0		38.1		0.0		60.0	
C																					
No	80	80.1	0.874	25	64.0	0.037	55	89.5	0.618	75.0	0.642	64.0	0.045	81.2	0.870	70.4	<0.001	64.0	0.037	75.0	<0.001
Yes	23	81.2		4	25.0		19	94.7		71.5		25.0		83.1		31.3		25.0		36.8	
N																					
No	98	80.5	0.646	29	58.6	NA	69	91.7	0.223	75.0	0.238	58.6	NA	83.2	0.088	64.0	0.001	58.6	NA	68.2	<0.001
Yes	5	80.0		0	NA		5	80.0		60.0				60.0		20.0		NA		20.0	
M																					
No	69	87.1	0.020	17	70.6	0.159	52	93.1	0.247	83.3	0.005	70.6	0.163	87.7	0.032	65.1	0.559	70.6	0.159	63.7	0.701
Yes	34	65.5		12	41.7		22	85.1		56.2		41.7		67.5		55.4		41.7		67.3	
VPEFR																					
No	48	85.3	0.135	16	75.0	0.019	32	92.3	0.597	83.7	0.023	75.0	0.024	89.6	0.115	83.2	<0.001	75.0	0.019	89.1	<0.001
Yes	55	76.3		13	38.5		42	90.2		66.2		38.5		75.7		43.2		38.5		47.1	
VPEFR2																					
No	71	85.5	0.027	22	68.2	0.030	49	94.9	0.066	83.3	0.001	68.2	0.037	91.3	0.002	74.2	<0.001	68.2	0.030	78.5	0.001
Yes	32	68.2		7	28.6		25	83.4		54.0		28.6		62.7		33.2		28.6		39.6	
CHIC‐HS																					
Very low	4	100.0	0.058	3	100.0	0.020	1	100.0	0.581	100.0	0.008	100.0	0.035	100.0	0.061	100.0	0.001	100.0	0.020	100.0	0.021
Low	33	89.0		7	85.7		26	90.1		86.9		85.7		87.1		85.9		85.7		86.2	
Intermediate	19	77.2		5	20.0		14	100.0		77.7		20.0		100.0		39.5		20.0		50.0	
High	47	73.6		14	50.0		33	87.3		62.3		50.0		69.0		50.7		50.0		54.0	

Abbreviations: AFP, alpha‐fetoprotein; CHIC‐HS, Children's Hepatic tumors International Collaboration‐Hepatoblastoma StratificationEFS, event‐free survival; OS, overall survival; PRETEXT, pre‐treatment extent of tumor; SCU, small cell undifferentiated; TFS, transplant‐free survival.

Survival outcomes did not differ according to sex and pathological subtypes. The 5‐year EFS rates were different according to age at diagnosis with superior outcomes among patients under 1 year of age (*P *= 0.025). Moreover, the OS rates did not differ according to age at diagnosis. In addition, the OS and EFS rates were significantly higher among patients with AFP in the first tertile at diagnosis compared with those in the second and third tertiles (*P *= 0.046 and *P *= 0.012, respectively).

The EFS (83.3% vs. 56.2%, *P *= 0.005) and OS (87.1% vs. 65.5%, *P *= 0.02) rates were significantly higher among patients without metastasis; however, the TFS rates were not different according to the presence of metastasis. In particular, the OS rates were improved significantly in the late period for patients with metastasis (41.7% to 85.1%, *P *= 0.024) or without metastasis (70.6%–93.1%, *P *= 0.014). Of 12 patients with metastasis, 5 patients died in the early period (3 patients with liver tumor progression and 2 patients with intrahepatic relapse). Moreover, of 22 patients with metastasis in the late period, 3 patients died from the progression of metastatic diseases.

### 3.4 Association of survival outcomes with the PRETEXT stage with annotation factors

**FIGURE 3:**
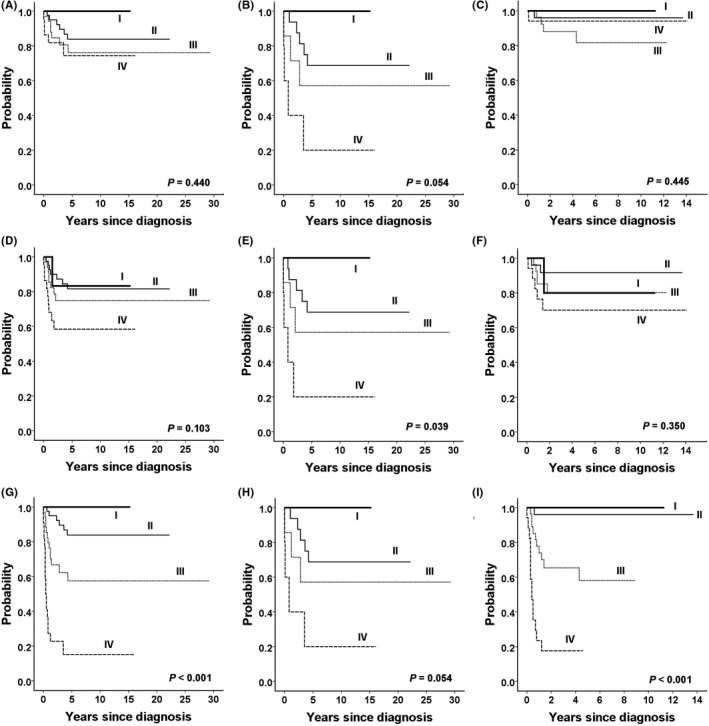
Outcomes according to the PRETEXT stage. Overall survival rates for the overall (A), early (B), and late (C) treatment cohorts. Event‐free survival rates for the overall (D), early (E), and late (F) treatment cohorts. Transplant‐free survival rates for the overall (G), early (H), and late (I) treatment cohorts. PRETEXT, pre‐treatment extent of tumor

Survival outcomes according to the PRETEXT stage are shown in Figure 3. During the entire study period, the OS and EFS rates did not differ according to the PRETEXT stage (*P *= 0.440 and *P *= 0.103, respectively); however, the TFS rates were significantly different (*P *< 0.001). Analysis of the outcomes according to the treatment era revealed that the PRETEXT stage was a significant prognostic factor associated with the EFS rates in the early period (*P *= 0.039) but not in the late period (*P *= 0.350). Similarly, the OS rates were different according to the PRETEXT stage with a marginal statistical significance in the early period (*P *= 0.054) but not in the late period (*P *= 0.445). The TFS rates were marginally different in the early period (*P *= 0.054) and significantly different in the late period (*P *< 0.001).

Evaluation of the association of the annotation factors with OS revealed that only the P and M factors were significant factors for poor OS over the entire study period. In the early period, the P, F, R, and C factors were significant predictors of poor OS; however, in the late period, the P factor was the only factor that remained significant. VPEFR positivity was not a significant factor in the entire study period (*P *= 0.135). Analysis stratified by the treatment era showed that VPEFR positivity was a significant factor in the early period (*P *= 0.019) but not in the late period (*P *= 0.597). VPEFR2 positivity was significant over the entire study period (*P *= 0.027) and in the early period (*P *= 0.030) and marginally significant in the late period (*P *= s0.066).

The causes of death among patients with the P factor were investigated. In the early period, four of five patients with the P factor died (two patients had primary refractory disease and two patients had relapses). In the late period, 3 of 14 patients with the P factor died (all of them had primary refractory disease).

Evaluation of the association of annotation factors with EFS revealed that the P, F, R, and M factors were significantly associated with EFS rates over the entire study period. In the early treatment period, the P, F, R, and C factors were significant predictors of EFS; however, in the late period, only the F and M factors were significant predictors of poor EFS, and the P, R, and C factors were no longer significant. Patients with positive VPEFR and positive VPEFR2 had significantly worse EFS rates over the entire study period. However, VPEFR positivity was not a significant factor in the late period (*P *= 0.115), whereas VPEFR2 positivity remained significant in the late period (*P* = 0.002).

In the early period, four of five patients with the F factor had events (three of them had refractory disease at the primary site and one patient had an intrahepatic relapse). In the late period, 9 of 30 patients with the F factor had events (3 of them had refractory disease at the metastatic site, 1 patient had refractory disease at the primary site, and 5 patients had a metastatic relapse).

Evaluation of the association of the annotation factors with TFS revealed that the V, P, F, C, and N factors were significant predictors over the entire study period, and these factors were still significant in the late period. In addition, VPEFR and VPEFR2 were significant predictors in both the early and late periods as well as the entire study period. The M factor was not a significant predictor of TFS regardless of the treatment period.

### 3.5 Association of survival outcomes with CHIC‐HS

**FIGURE 4:**
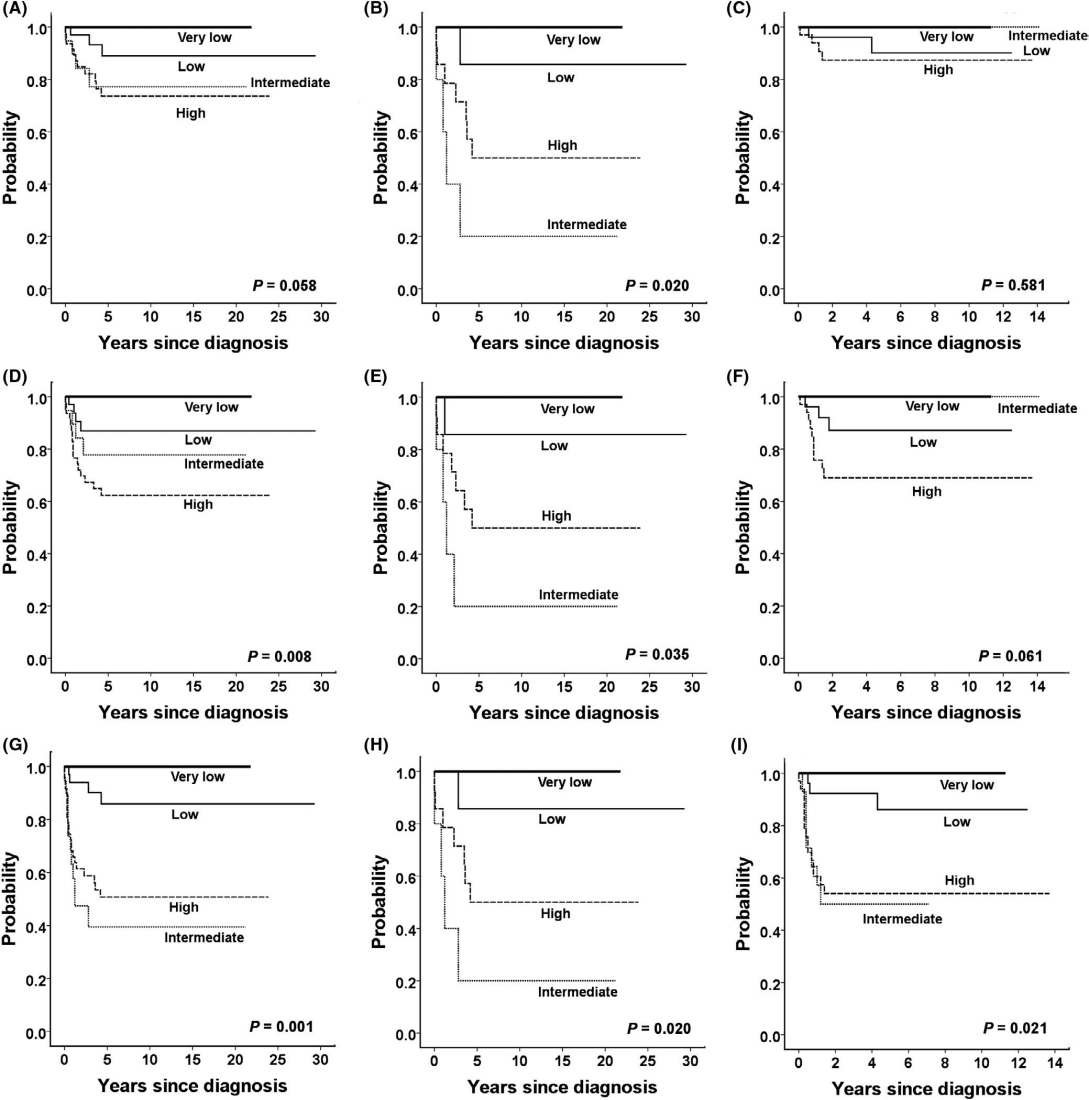
Outcomes according to the CHIC‐HS system. Overall survival rates for the overall (A), early (B), and late (C) treatment cohorts. Event‐free survival rates for the overall (D), early (E), and late (F) treatment cohorts. Transplant‐free survival rates for the overall (G), early (H), and late (I) treatment cohorts. CHIC‐HS, Children’s Hepatic tumors International Collaboration‐Hepatoblastoma Stratification

Survival outcomes according to CHIC‐HS are shown in Figure 4. Groups with a higher risk had significantly worse EFS rates over the entire study period (*P *= 0.008). Analysis stratified by the treatment period showed that CHIC‐HS was associated with EFS rates in the early period (*P *= 0.035) and marginally associated with EFS rates in the late period (*P *= 0.061). The prognostic significance of CHIC‐HS for OS was approaching marginal significance over the entire study period (*P *= 0.058). CHIC‐HS was associated with OS rates in the early period (*P *= 0.02) but not in the late period (*P *= 0.581). CHIC‐HS was associated with TFS rates in both the early and late periods as well as the entire study period.

## 4 DISCUSSION

This study was a retrospective study evaluating the effect of advances in surgical techniques and intensification of chemotherapy on prognostic factors and survival outcomes in hepatoblastoma. In the study cohort, both the EFS and OS rates were significantly improved in the late period, whereas the TFS rates remained unchanged. The PRETEXT stage was significant or marginally significant for EFS and OS in the early period but not in the late period. In particular, the outcomes of patients with higher PRETEXT stages with positive annotation factors and distant metastasis were significantly improved. We showed that the clinical implications of the PRETEXT stage with annotation factors could vary depending on the advances in treatment modalities. We also showed that CHIC‐HS had significant clinical implications in a real‐world setting.

PRETEXT staging at diagnosis is crucial for the risk stratification of patients with hepatoblastoma. Several trials have confirmed that the PRETEXT stage is a strong predictor of OS for patients with hepatoblastoma.^5^, ^10^, ^11^ In the Intergroup CCG/POG study INT‐0098, the OS rates of patients with PRETEXT IV hepatoblastoma were as low as 30.9%.^10^ However, a recent SIOPEL study showed that the OS rates of PRETEXT IV patients were increased from 61% in SIOPEL‐2 to 88% in SIOPEL‐4.^4^ In addition, the JPLT‐2 trial showed an improved OS rate of 67% among patients with PRETEXT IV disease compared with 50.3% in the JPLT‐1 trial.^5^, ^22^ In the present study, survival outcomes were significantly associated with the PRETEXT stage in the early cohort but not in the late cohort. In particular, the OS rates of patients with PRETEXT IV were greatly improved from 20.0% to 94.1% in the late period. However, the TFS rates were still significantly associated with the PRETEXT stage in both the early and late periods. This finding demonstrated that LT had a strong effect on survival outcomes, and the clinical implication of the PRETEXT stage should be reappraised considering recent advances in surgical techniques. The results of the present study suggest that PRETEXT staging is vital for surgical planning and risk stratification; however, it cannot be regarded as a predictor of the final survival outcome.

The CHIC database indicates the significant association of the V, P, E, F, R, and M factors with EFS.^13^ In the present study, among the annotation factors, the F factor was significantly associated with EFS in both the early and late periods, whereas the P, R, and C factors were not associated with EFS in the late period. This finding suggests that the P, R, and C factors can be overcome by an advanced surgical approach and intensified chemotherapy. In particular, events among patients with the F factor were mostly intrahepatic in the early period but mostly extrahepatic in the late period. This may explain the persistence of the F factor as a significant predictor of EFS even though multifocal intrahepatic tumors could have been controlled with extended hepatectomy or LT.

OS was associated with the P factor rather than the F factor as metastatic relapse in patients with the F factor could be treated by metastasectomy and salvage chemotherapy. The OS rates of patients with the P factor were improved significantly from 20% to 76.6% in the late period; however, patients with the P factor had a significantly worse outcome. The causes of death were mostly primary refractory disease and not relapse. This finding suggests that novel therapeutic approaches for primary refractory disease are needed to improve the cure rate of hepatoblastoma.

TFS was associated with the V, P, F, and C factors regardless of the treatment period; however, some of these factors were no longer associated with OS or EFS in the late period. The results suggest that anatomical risk factors in hepatoblastoma could be overcome with LT, which can lead to improved final survival outcomes. Interestingly, TFS did not differ according to the presence of metastasis. These results suggest that LT is a viable option for patients with advanced hepatoblastoma and lung metastasis. It is noteworthy that this finding is in contrast to that for adult hepatocellular carcinoma, where the outcome of LT is significantly poor for patients with a large tumor or gross vascular invasion or distant metastasis.^23^, ^24^

Distant metastasis at initial diagnosis has been reported as one of the most important prognostic factors associated with poor outcomes.^5^, ^8^, ^13^, ^25^ In the late period, metastasis was significantly associated with poor EFS but not OS. The OS rates of patients with metastasis were improved significantly from 28.6% to 83.4%, which was comparable to the outcome of the 3‐year OS 79% of the SIOPEL‐4 trial.^4^ The causes of death among patients with metastasis were mostly intrahepatic disease in the early era; however, metastatic disease was the main cause in the late period. This finding suggests that control of the primary disease should be achieved first, and primary tumor control combined with metastasectomy and intensified chemotherapy could significantly improve the outcome of patients with distant metastasis.

Based on CHIC‐HS, a linear trend of decreasing EFS was observed in the overall cohort. This result suggests that CHIC‐HS may be applied in an actual clinical setting in a single institution. In particular, in the early cohort, the intermediate‐ and high‐risk groups showed inferior EFS; however, in the late cohort, the EFS of the intermediate‐risk group was greatly improved, whereas that of the high‐risk group remained inferior. In the late period, the OS rates were no longer different according to CHIC‐HS because the outcomes of patients with metastasis and advanced PRETEXT stages were improved. However, in contrast, a retrospective registry study in Hong Kong from 1996 to 2014 reported that the CHIC risk groups significantly predicted EFS and OS.^26^ The present study demonstrated that recent advances in the treatment of hepatoblastoma could reduce the association of CHIC‐HS with OS outcomes.

There are several limitations in the present study. This was a single study over a long period. However, we were able to investigate the details of treatments, which demonstrated the advances in treatment modalities (especially changes in surgical options) over the long term, and determine the implication of prognostic factors in an actual clinical setting. In addition, patients in the late period had a shorter follow‐up period. However, most events and deaths usually occur within 3 years of diagnosis; thus, a longer follow‐up period would not change the outcomes significantly.

In conclusion, survival rates were significantly improved in children with hepatoblastoma, especially in those with advanced PRETEXT stages with positive annotation factors and in a high‐risk CHIC‐HS group, since the introduction of effective chemotherapy and advanced surgical approaches, including LT. These advances can affect the traditional risk factors with new clinical implications in recent years. Therefore, it is necessary to reappraise the clinical significance of the PRETEXT stage and its annotation factors and CHIC‐HS system considering advances in surgical techniques and accessibility to these surgical modalities. Efforts should be focused on introducing a risk‐stratified approach and refining the indications of LT and intensified chemotherapy to reduce the late effects of treatment.

### 4.1 Ethics statement

The study was approved by the institutional review board of Asan Medical Center (No. 2020–1455) and the requirement for informed consent was waived due to the retrospective design.

## CONFLICT OF INTEREST

The authors declare that they have no conflict of interest.

## AUTHOR CONTRIBUTIONS

**Kyung‐Nam Koh:** Conceptualization, data curation, formal analysis, writing‐o‐riginal draft, and writing‐review and editing. **Jung‐Man Namgoong:** Conceptualization, data curation, methodology, formal analysis, and writing‐review and editing. **Hee Mang Yoon:** Conceptualization, data curation, methodology, formal analysis, and writing‐review and editing. **Young Ah Cho:** Methodology and writing‐review and editing. **Se Hoon Choi:** Data curation and writing‐review and editing. **Juhee Shin:** Software and visualization. **Sung Han Kang:** Data curation and writing‐review and editing. **Jin Kyung Suh:** Data curation and writing‐review and editing. **Hyery Kim:** Investigation and writing‐review and editing. **Seak Hee Oh:** Data curation and writing‐review and editing. **Kyung Mo Kim:** Supervision and writingreview and editing. **Dae Yeon Kim:** Supervision and writing‐review and editing. **Ho Joon Im:** Supervision and writing‐review and editing.

## Data Availability

The datasets used and/or analyzed during the current study are available from the corresponding author upon reasonable request.
